# Bioinformatic Tools for NGS-Based Metagenomics to Improve the Clinical Diagnosis of Emerging, Re-Emerging and New Viruses

**DOI:** 10.3390/v15020587

**Published:** 2023-02-20

**Authors:** Marta Ibañez-Lligoña, Sergi Colomer-Castell, Alejandra González-Sánchez, Josep Gregori, Carolina Campos, Damir Garcia-Cehic, Cristina Andrés, Maria Piñana, Tomàs Pumarola, Francisco Rodríguez-Frias, Andrés Antón, Josep Quer

**Affiliations:** 1 Liver Diseases-Viral Hepatitis, Liver Unit, Vall d’Hebron Institut de Recerca (VHIR), Vall d’Hebron Hospital Universitari, Vall d’Hebron Barcelona Hospital Campus, Passeig Vall d’Hebron 119-129, 08035 Barcelona, Spain; 2 Centro de Investigación Biomédica en Red de Enfermedades Hepáticas y Digestivas (CIBERehd), Instituto de Salud Carlos III, Av. Monforte de Lemos, 3-5, 28029 Madrid, Spain; 3 Biochemistry and Molecular Biology Department, Universitat Autònoma de Barcelona (UAB), Campus de la UAB, Plaça Cívica, 08193 Bellaterra, Spain; 4 Microbiology Department, Vall d’Hebron Institut de Recerca (VHIR), Vall d’Hebron Hospital Universitari, Vall d’Hebron Barcelona Hospital Campus, Passeig Vall d’Hebron 119-129, 08035 Barcelona, Spain; 5 Microbiology Department, Universitat Autònoma de Barcelona (UAB), Campus de la UAB, Plaça Cívica, 08193 Bellaterra, Spain; 6 Department of Basic Sciences, Universitat Internacional de Catalunya, Sant Cugat del Vallès, 08195 Barcelona, Spain

**Keywords:** NGS, deep-sequencing, viruses, metagenomics, zoonosis, diagnostic tools

## Abstract

Epidemics and pandemics have occurred since the beginning of time, resulting in millions of deaths. Many such disease outbreaks are caused by viruses. Some viruses, particularly RNA viruses, are characterized by their high genetic variability, and this can affect certain phenotypic features: tropism, antigenicity, and susceptibility to antiviral drugs, vaccines, and the host immune response. The best strategy to face the emergence of new infectious genomes is prompt identification. However, currently available diagnostic tests are often limited for detecting new agents. High-throughput next-generation sequencing technologies based on metagenomics may be the solution to detect new infectious genomes and properly diagnose certain diseases. Metagenomic techniques enable the identification and characterization of disease-causing agents, but they require a large amount of genetic material and involve complex bioinformatic analyses. A wide variety of analytical tools can be used in the quality control and pre-processing of metagenomic data, filtering of untargeted sequences, assembly and quality control of reads, and taxonomic profiling of sequences to identify new viruses and ones that have been sequenced and uploaded to dedicated databases. Although there have been huge advances in the field of metagenomics, there is still a lack of consensus about which of the various approaches should be used for specific data analysis tasks. In this review, we provide some background on the study of viral infections, describe the contribution of metagenomics to this field, and place special emphasis on the bioinformatic tools (with their capabilities and limitations) available for use in metagenomic analyses of viral pathogens.

## 1. Introduction

Viruses, particularly those with an RNA genome, are characterized by high variability, a feature that facilitates easy adaptation to changing environments. When viral genomes isolated from samples of infected patients are sequenced, one sees a mixture of different but closely related genomes that undergoes continuous changes over time, mainly because the RNA-dependent RNA polymerases essential for replication often lack proofreading mechanisms [1]. This complex mixture of closely related genomes is known as a viral quasispecies, and the continuous changes in quasispecies composition result from competitive selection [2] and cooperation [3] between arising mutants. 

The abundance or frequency with which a specific genome is found in the viral quasispecies depends on its fitness (replication efficacy) and other known and unknown viral and host factors [2,3]. As a consequence of the multiple variants produced during replication, the virus may obtain certain advantages in addition to adaptability: reduced sensitivity to antiviral therapy, escape from the immune response and vaccine protection, and the possibility to invade new niches. Hence, because of their inherent characteristics, “new” viruses with pathogenic capability in humans can arise. 

Our experience with SARS-CoV-2 has clearly shown that viruses cannot be walled-off and that human travel bans are ineffective for preventing expansion of an infection [4]. A better strategy to face the emergence, re-emergence, or appearance of new infectious genomes would be prompt detection and identification, so that specific tools can be applied to tackle the threat. Immediate implementation of control measures at the start of an infection, when there are only a few cases, would have the greatest success in controlling transmission. 

Prompt detection implies an accurate disease diagnosis, but the data show that 50% to 60% of acute infections have an unidentified etiology, and 60% to 80% of meningitis/encephalitis, 50% of acute gastroenteritis, 20% of hemorrhagic fever, and 15% to 25% of acute respiratory infections are incorrectly diagnosed [5,6,7,8,9]. As examples of this situation, in the California encephalitis project including 1570 patients, the cause of encephalitis was not identified in 63% of patients [5], and this figure rose to 80% in a study in France [6]. Diagnostic failure can result in delayed and ineffective treatments, with increases in mortality and excessive health expenditure. Hence, correct identification of the infectious agents associated with human disease is a priority. 

Currently available diagnostic tests are limited for detecting new pathologic agents. Identification of a virus during acute infection is of enormous value, but current techniques do not enable us to rule out the presence of other coinfecting agents that may be pathogenic. As a large number of pathogens can cause a syndromic infection, a high throughput method such as next-generation sequencing (NGS) adapted to simultaneously detect any pathogen present could be more advantageous than the use of a large number of individual tests based on current methods [10,11,12] Furthermore, NGS [13] and metagenomics could potentially be used in the future in a wide range of diseases, such as cancer or gastrointestinal infections, in which the findings obtained could lead to the identification of new treatment targets [14,15]. 

The sensitivity to detect a virus in a bodily fluid is determined by three factors: the concentration of the virus in the clinical sample, the amount of total RNA and DNA (which compete with what we intend to detect), and the analytical sequencing depth. Sequencing depth can be resolved using high throughput equipment, but the viral concentration cannot be improved unless large amounts of sample are accessible. To overcome the problem caused by background, enrichment of the sample’s viral content is required. This is achieved by removing ribosomal RNA (rRNA), by DNaseI treatment to significantly reduce the amount of free DNA, by using panels with specific capture probes, or by using specific primers in multiplex amplification. Research still has a long way to go to improve pre-sequencing methods to increase the sensitivity and efficiency of current techniques for enhanced clinical diagnosis of emerging, re-emerging, and new viruses. High-throughput NGS technologies based on metagenomics could be the answer to overcome these limitations.

Next-generation sequencing based on metagenomics is a powerful technique to confront the challenge of genetic identification and characterization of known and unknown viral genomes in a large variety of human cell and tissue samples. Metagenomics can be used to design tests based on PCR, to develop mRNA- and DNA-based vaccines, to design direct-acting compounds that block a pathogen’s specific functions, to study variability and enable correct classification of an infecting agent, and to identify genetic markers associated with the severity of an infection, antigenicity, and the evolution of new variants [16,17,18,19,20,21].

## 2. What Is Metagenomics?

Metagenomics is a field of NGS that enables identification of microbial communities, and genetic detection, identification, and characterization of disease-causing agents. It has proven to be a key element in genetic characterization of viruses and has led to discoveries that would not have been accomplished using traditional culturing techniques [22]. Current molecular assays target a limited number of pathogens using specific primers or probes, whereas metagenomics can approach all DNA and RNA molecules present in a sample, enabling analysis of the corresponding host genome and its collection of microbes [23]. The capability of metagenomics to detect any genome, including bacteria, viruses, parasites, and fungi in a human sample is of great interest for the diagnosis of infectious diseases. Metagenomic approaches have also been applied to several other research areas: environmental studies (e.g., marine samples, soil, sewage, farm dust) [24,25,26,27,28]; viral infection in Bronze Age human samples, 7000 years old [29,30]; characterization of the human gut microbiome in health, disease, and forensic investigation [31,32,33,34]; clinical studies [23,35]; and discovery of new viral pathogens such as SARS-CoV-2 [36,37].

### 2.1. What Does Metagenomics Involve?

Metagenomic analyses are performed with random primers that contain every possible combination of nucleotides. This results in the presence of all possible hexamers and allows primers to bind to any RNA or DNA molecule in a mixture of genomes. Once amplified, the PCR product can be loaded onto any NGS platform to obtain millions of short sequences (reads) smaller than 600 bases, but usually <300 bases -Illumina (San Diego, CA, USA), ThermoFisher Scientific (Waltham, MA, USA), MGI (Shenzhen, China), Complete Genomics (San José, CA, USA); suppliers- or long reads of around 1 kb or more -PacBio (Menlo Park, CA, USA), Oxford Nanopore Technologies (Oxford, UK); manufacturers-, and analyzed through bioinformatic techniques [38]. This methodology has several desirable advantages. It does not require any prior knowledge of the genomes under study for primer or probe design and is useful for de novo sequencing or resequencing. It enables identification of all pathogens present in a wide variety of samples, including cerebrospinal fluid, sputum, serum, plasma, stool, amniotic fluid, and many others [39,40,41,42,43], and can identify the major genomes of viral populations in epidemiological studies, outbreaks, and phylogenetic analyses. 

However, the technique also has strong disadvantages. It requires a large quantity of genomic starting material (e.g., a high viral load) and the analysis is quite complex. The capability to amplify any DNA or RNA genome at random can lead to an underrepresentation or even a loss of minority genomes (low sensitivity), as DNA from the host genome and commensal microorganisms are also amplified. Another important factor to take into account is the risk of contamination during sample collection and the analytical process, which can complicate interpretation of the results. RNA contamination or cross-contamination can be managed by including several controls and quality check points [44]. For example, negative controls consisting of RNase-free water could be added to detect undesirable contamination or human error [44,45]. An additional approach is to include controls in the data analysis to identify contaminants [44].

Briefly, the laboratory protocols for viral metagenomics include sample collection, virus enrichment, DNA/RNA extraction, and library preparation for sequencing. Sample collection and processing varies considerably and depends on the type of biological fluid to analyze [46]. After obtaining the free virions in suspension, several viral enrichment techniques can be applied, as will be reviewed later. Nucleic acid extraction is mainly based on the use of silica spin columns, including lysis buffers containing chaotropic salts and detergents, and optional use of DNA or RNA carriers [47]. Finally, library preparation involves amplification enrichment after fragmentation using kits such as the Illumina TruSeq RNA Library Prep or Nextera XT DNA [44]. The proper choice among the various available options in each step is key to enhancing the quality of the results for each specific aim.

Of note, recent advances in sequencing technology have enabled long-read sequencing (LRS). With LRS, reads longer than 10 kbp can be generated [48], facilitating the process of mapping against a reference genome and identifying different species in complex samples. Furthermore, the evolution of single-molecule real-time (SMRT) technology through circular consensus sequencing (CCS) has overcome the problem that LRS typically has with base accuracy per read [49]. Long-read sequencing can be performed using various platforms, such as PacBio and Oxford Nanopore Technologies.

### 2.2. Alternatives to Metagenomics

NGS for metagenomics, whole-genome sequencing (WGS), and targeted deep-sequencing are currently the best tools available for genetic identification and characterization of viruses. With the use of these techniques we can correctly classify a virus, determine its variability, identify viral genetic markers associated with virulence, and consider antigenicity and susceptibility to antivirals based on pre-existing knowledge, when available [16,17,19].

### 2.3. Enrichment of Viruses in Metagenomic Samples

One of the main issues in virome sequencing is related to the viral titer present in the sample. Usually, viruses account for only a small percentage of the total of genomes present, with most of them belonging to other types of organisms. Depending on sample type, several methods have been used to concentrate the virions present [50]. Viruses in water can be concentrated based on their surface charge, using flocculation-precipitation methods or negative/positive membrane filters [51,52]. Size-based techniques can be used for viral concentration in both water and cell culture supernatants [53,54]. Selection of cell-free DNA (cfDNA) in plasma based on size can enrich viral DNA, as cfDNA size varies according to the species [55]. Finally, the ViroCap project has created a sequence capture panel for viral RNA from 34 families of viruses, notably increasing viral coverage [56].

## 3. Available Bioinformatic Tools

A number of tools designed for various purposes are now available for use in metagenomics [57,58]. Our focus in this review is to examine analytical tools that facilitate identification of emerging, re-emerging, and new viruses in samples having an animal origin.

There are five main steps in metagenomic assemblies to identify viruses: quality control (QC); quality trimming; read assembly and assembly QC (which are optional); and taxonomic classification of assemblies with two different aims—the identification of known viruses that have already been sequenced and the identification of viruses that have not been sequenced or are unknown. Metagenome binning is an additional step that can be performed before taxonomic profiling. The aim of binning is to cluster assembled sequences according to their origin.

### 3.1. Bioinformatic Tools for Data QC

The first step in metagenomics would be to perform sequence QC, as it is essential to remove technical errors from the analysis. The main objective of this step is to pre-process the data to eliminate undesirable adapter sequences, excessively short reads, low-quality reads or nucleotides, and others that may be present. Several programs can be used in this step, depending on the data being analyzed (Table 1).

For short-read sequencing data, QC can be performed with FastQC (https://www.bioinformatics.babraham.ac.uk/projects/fastqc/, accessed on 23 December 2022), which checks the quality of the data and generates a report summarizing its metrics. The same type of report is available with other QC programs, such as MultiQC [59], which has the same functionality as FastQC with one main difference, it can be applied to several fastQ files simultaneously and generate one main report for all the files provided.

For the preprocessing of long-read sequencing data, longQC [60] or MinionQC [61] can be used the determine sequence quality. These have been applied to data obtained with Nanopore’s MinION and other long-read sequencers.

### 3.2. Bioinformatic Tools for Data Pre-Processing

After having identified technical errors within the metagenomic data, such as excessively short reads, low-quality reads, and adapters, the next step is to eliminate them to avoid false positives and negatives. In addition, removal of undesirable sequences will decrease computational time and cost in the following steps. Several tools can be used for the removal of unwanted sequences or nucleotides, depending on the pre-processing aim and what has been identified during the QC step.

#### 3.2.1. Tools for Quality Trimming

In metagenomics and in most RNA sequencing methods, the first step would be to trim unwanted elements identified through the data QC programs (Table 1). The main program for this step used in short-read sequencing is Trimmomatic, a bioinformatic tool designed to remove low-quality reads and adapters [62]. Another possibility is cutadapt, which identifies and removes adapters and other sequence types [63] (Table 2).

Long-read sequencing fastq files can be trimmed with other programs (Table 2). For example, NanoPack [64] can be used to process data from long-read sequencing and to visualize QC results. SequelTools [65], which has the same functionality as Nanopack, is another option.

#### 3.2.2. Tools for Filtering Untargeted Reads

The second filtering step is to eliminate reads of no interest, which can be derived from various sources. When targeting viral reads, we would have to remove reads belonging to the host genome and contaminants. This is a key step to decrease the execution time and computational expense in taxonomic classification of dataset sequences. In addition, it reduces false positives and can prevent assembly of chimeric virus–host sequences [66]. Several strategies are commonly used to remove these sequences from the data.

For example, read mappers can be applied to remove all sequences mapping a selected reference genome, which could belong to the host genome or possible contaminants. For short reads, mappers such as BWA [67], bowtie2 [68], and BBMap [69] are available (Table 3). Other tools such as FastQ-Screen (https://www.bioinformatics.babraham.ac.uk/projects/fastq_screen/, accessed on 23 December 2022) can identify sequences belonging to specific genomes. These programs determine the proportion of host genome or other specific contaminant sequences against target reads.

The filtering of long reads can be performed by some of the above-mentioned read mappers, such as BWA [70] and BBMap [69], or more specific ones, such as minimap2, which was particularly designed for long-read sequencing data [71].

Alternative approaches are available to reduce the number of unwanted sequences. Certain bioinformatic tools have been programmed to identify specific sequences belonging to specific taxa. In this case, once the reads have been trimmed, the sequences are passed through a filtering program that only selects reads with certain features. An example is VirusHunter (https://bio.tools/virushunter, accessed on 23 December 2022), used to identify viral sequences in NGS data. RINS is another option, as it has been designed to identify non-human sequences [72] and can filter out reads from a human source.

In some situations, other RNA sequence types such as ribosomal (rRNA), mitochondrial (mtRNA), or messenger (mRNA) types from untargeted taxa may have to be removed from metagenomic data [66]. RiboDetector (https://github.com/hzi-bifo/RiboDetector, accessed on 23 December 2022) can be used for this purpose, as it is designed to identify rRNA, which can thus be filtered out to improve the following analyses.

Another approach involves taxonomic profiling of the reads before assembly. With this strategy, sequences other than those belonging to viruses can be filtered out, and viral sequences retained for further analysis. This option can be carried out with taxonomic classifiers, such as kraken2 [73] and kaiju [74], which will be further explained later.

### 3.3. Bioinformatic Tools for Assembly

#### 3.3.1. Tools for Short-Read Assembly

To perform taxonomic assignment and identify the viruses present, we must first restore the metagenomes. This implies the generation of contigs, sets of sequences that have been overlapped to provide a longer, continuous sequence.

The main type of assembly used in metagenomics is called de novo genome assembly. This can be performed by overlap layout consensus, based on overlapping the read ends, or with various algorithms, such as de Bruijn graphs, which split the sequences into smaller fragments, called k-mers, thereby reducing the time and computational effort needed for analysis [75]. In addition, several programs are available to perform de novo assembly, for example, MEGAHIT [76], a bioinformatic assembler tool optimized for metagenomes, or metaSPADES [77] and IDBA-UD [78], which are also optimized for metagenomes (Table 4).

Nonetheless, the assembly of sequences is quite complex. In metagenomic analyses, where there is a huge volume of data and unequal representation of the microbial community, genomes which have a small presence are usually underrepresented [79]. Furthermore, de novo assembly can generate errors, as a single sample can contain sequences from very similar organisms.

Another strategy, reference-based assembly, can also be used in metagenomics. However, appropriate reference genomes may not be available in all cases, and this approach does not allow the identification of new viruses, or viruses that have not been sequenced previously.

#### 3.3.2. Tools for Long-Read Assembly

Various programs have been designed to assemble long reads. These are specific for this kind of technology and take into account the higher error rate associated with these reads. Some examples are metaFlye [80], Canu [81], and NECAT [82]. These tools can be used with data from various techniques, from Nanopore sequencing to PacBio, even in high-fidelity reads (Table 4).

#### 3.3.3. Tools for Hybrid Assembly

As was mentioned above, short reads are known to have very low error rates, but they cannot be used in some cases, such as in the assembly of highly variable regions. Long reads can provide continuous coverage of very long regions, but they have high error rates [55]. The optimal situation would be to combine both these features: coverage of long regions with the reliability of short reads.

Accordingly, various programs have been developed to perform hybrid assembly, specifically in metagenomic studies. OPERA-MS [83], implemented with the de Bruijn graph algorithm, works by first assembling short reads to produce contigs, and then mapping both long and short reads to the contigs. Once this is completed, long reads are then used to connect the contigs. Finally, the contigs are clustered according to their genomic distance and difference in coverage [83]. HybridSPADES [84] is another program that can perform hybrid assembly. It is based on the de Bruijn graph algorithm, which first constructs an assembly graph using short reads, and then maps long reads to this first assembly, closing the gaps and resolving repeats using long reads [84]. HASLR [85] and Wengan [86] are additional tools that perform assembly in a manner similar to HybridSPADES (Table 4).

### 3.4. Bioinformatic Tools for Quality Control of Metagenome Assembly

Once the metagenome has been assembled, the quality of the assembly should be determined. The tools for this purpose can be classified into two main categories: those that require reference genomes for QC such as MetaQUAST [87], which uses references to calculate statistics for the assembly; and those that do not need references, such as DeepMAsED [88], which uses machine learning to identify misassemblies, or REAPR [89], which computes basic statistics using paired-end reads mapped to the assembly (Table 5). In general, it can be difficult to work with references in metagenomic studies, as reference genomes are often unavailable or of very poor quality.

BUSCO [90] is another assembly evaluator, in this case working with program-based reference datasets to assess completeness of the metagenome [90]. CheckM [91] can also be used for assembly evaluation. It provides estimates of the completeness of the genome, such as GC content. Finally, VALET (https://github.com/marbl/VALET, accessed on 23 December 2022) can be applied to detect misassemblies in metagenomic data, as it can bin contigs by coverage and avoid false positives and false negatives due to uneven coverage depth [57,92]

Quality control statistics for genome assembly may not be appropriate for assessment of metagenome assemblies. For example, the N50 measure describes the quality of the assembly based on the minimum contig length, which represents half the genome. Nonetheless, this is not representative of metagenomic data, which contain a wide range of genomes having different sizes. Thus, there is a need to develop QC statistics for metagenome assembly. As an alternative to the programs mentioned, reads could be mapped to contigs to determine the quality of the assembly. This would enable a more general assembly check.

An essential step to identify viruses in metagenomic analyses is to perform taxonomic profiling. There are two main methods to achieve this task: the first is to classify reads according to taxonomies, and the second is to establish taxonomic groups by contigs. Both methods have advantages and disadvantages. In taxonomic profiling with contigs (i.e., with assembled reads), classification is conducted with longer sequences. However, there is a risk that some contigs might be chimeric. Taxonomic profiling with reads is less statistically significant; the sequences are shorter, although a larger number of sequences are analyzed [93]. This approach could provide a more diverse result, but the computational expense would be higher.

### 3.5. Tools for Identification of Known Viruses

Taxonomic profilers use reference databases to compare reads/contigs with given sequences. Some profilers have been implemented using k-mers, such as kraken2 [73], bracken [94], CLARK [95], and Centrifuge [96] (Table 6). There are also protein-based programs, which first translate sequences to enable a comparison with reference protein databases. For example, kaiju can input reads or contigs, which are translated to proteins and then queued to the system to find matches. DIAMOND [97] and MMseqs2 [98] are additional protein-based programs. Tools such as MetaPhlAn4 [99], IGGsearch [100], and GOTTCHA [101] involve the use of gene markers to align sequences to gene marker-related databases.

Other programs are based on algorithms, such as BLAST or DUDes [102], which execute a new algorithm using the DUD (Deepest Uncommon Descent) strategy [103]. Some bioinformatic tools have been specifically programmed to study the virome, including VirusTaxo [104], Metavir2 [105], and DeepVirFinder [106], whose main algorithm is based on convolutional neural networks (CNN).

On the other hand, some tools, such as MetaPhlAn4 [99] and MCP (Microbiota Community Profiler), contain sequences from unidentified metagenomics-assembled genomes. This allows the identification of viruses that are not available in corresponding databases. However, MCP can only be used to identify bacterial, archaeal, eukaryotic, and viral sequences in microbiota studies [107].

It is important to keep in mind that each taxonomic profiler performs differently, and that a variety of algorithms and reference databases are used. This multiplicity can lead to differing results according to the program applied, and a wide range of time and computational expenses. K-mer-based taxonomic profilers seem to be the most computationally efficient, although they have high memory demands. Marker-based classifiers have lower memory requirements, but they can only classify reads or contigs from specific regions. Alignment-based bioinformatic programs are more computationally expensive than the others [107].

#### Tools for Identification of Novel Viruses

Programs to identify viral sequences without the need for any reference are now available. VirSorter [108] and VirFinder [106] are two such bioinformatic tools. VirFinder [106] is a k-mer-based R package that can identify viral contigs with good predictive accuracy, whereas VirSorter can identify novel viral sequences in a diverse microbial dataset [105].

Specific taxonomic profilers, such as MCP [107], that include unidentified metagenome-assembled genomes (MAGs) retrieved from microbiota studies, enable the detection of sequences that are found in other metagenomic datasets, but have not been uploaded to the main databases. However, not many profilers can do this. It would be of great value to upload unidentified MAGs to provide a consortium of unrecognized sequences and enable a more efficient and effortless analysis.

Despite these advances, better information and tools are needed to enable recognition of emergent and new viruses whose sequences are not present in reference databases. Robust approaches and programs should be developed to detect new viral sequences in metagenomic analyses. As an example, once the metagenome has been assembled, a possible strategy could be to classify sequences according to their proportion of tetranucleotides. This would allow similar sequences to be clustered together, possibly indicating a similar origin. However, this method would need to be reinforced by analysis of the sequence characteristics, such as ORFs and protein-coding sequences. Several available bioinformatic programs have been designed to do this, such as Prodigal [109], which can find protein-coding sequences within contigs.

### 3.6. Bioinformatic Tools for Contig Binning

An optional step, contig binning, can be performed before taxonomic profiling. The main goal of contig binning is to cluster contigs according to species, as these sequences usually do not cover the whole genome [57]. Several tools using different core algorithms for short-read sequencing can be used for this purpose (Table 7). One example is CONCOT [110], a program that allows the clustering of metagenomic contigs according to nucleotide composition and coverage data. Another is GraphBin [111], which uses the assembly’s connectivity information to cluster contigs.

Metagenomic binning is not restricted to contigs. It can also be conducted with reads, specifically, long reads, and with the use of MEGAN-LR [112], BusyBee [113], or LRBinner [114] (Table 6).

## 4. Conclusions

Numerous recent advances have been achieved in the field of metagenomics. This technique can aid in the discovery of new viruses, prediction of outbreaks, and diagnosis of certain diseases for clinical purposes, among others. The rapid evolution of long-read sequencing platforms can benefit metagenomic analyses by producing more reliable results. In metagenomics in virology, appropriate sample processing is important to detect viromes without underrepresentation.

Despite this progress, further developments are needed. For example, consensus guidelines would be of great value to promote proper data analysis. As is seen in this review, numerous programs using various approaches are available to analyze this type of data, and several pipelines have been developed to enable faster and easier analysis. However, these are based on very different processes and there is still a lack of consensus regarding their performance for various tasks. Another key undertaking is to maintain the related databases updated, as these are essential for identification of taxa within the data.

## Figures and Tables

**Table 1:** Bioinformatic programs for data quality control in short-read and long-read sequencing. All websites/GitHub Repository links were accessed on 23 December 2022.

Program	Type of Data	Website/GitHub Repository
FastQC	Short reads	https://www.bioinformatics.babraham.ac.uk/projects/fastqc/
MultiQC	Short reads	https://multiqc.info
LongQC	Long reads	https://github.com/yfukasawa/LongQC
MinionQC	Long reads	https://github.com/roblanf/minion_qc

**Table 2:** Bioinformatic programs for data trimming in short-read and long-read sequencing. All websites/GitHub Repository links were accessed on 23 December 2022.

Program	Type of Data	Website/GitHub Repository
Trimmomatic	Short reads	http://www.usadellab.org/cms/?page=trimmomatic
Fastp	Short reads	https://github.com/OpenGene/fastp
Cutadapt	Short reads	https://cutadapt.readthedocs.io/en/stable/
SOAPnuke	Short reads	https://github.com/BGI-flexlab/SOAPnuke
NanoPack	Long reads	https://github.com/wdecoster/nanopack
SequelTools	Long reads	https://github.com/ISUgenomics/SequelTools

**Table 3:** Bioinformatic mappers for filtering out untargeted or contaminant sequences in short-read and long-read sequencing data. All websites/GitHub Repository links were accessed on 23 December 2022.

Program	Type of Data	Website/GitHub Repository
BWA	Short reads, long reads	https://bio-bwa.sourceforge.net
Bowtie2	Short reads	https://github.com/BenLangmead/bowtie2
BBMap	Short reads, long reads	https://jgi.doe.gov/data-and-tools/software-tools/bbtools/bb-tools-user-guide/bbmap-guide/
Minimap2	Long reads	https://github.com/lh3/minimap2

**Table 4:** Bioinformatic tools for metagenome assembly for short-read, long-read, and hybrid assemblies. All websites/GitHub Repository links were accessed on 23 December 2022.

Program	Read Length	Algorithm	Website/GitHub Repository
MEGAHIT	Short reads	De Bruijn graph	https://github.com/voutcn/megahit
metaSPADES	Short reads	De Bruijn graph	https://github.com/ablab/spades
IDBA-UD	Short reads	De Bruijn graph	https://i.cs.hku.hk/~alse/hkubrg/projects/idba_ud/
MetaVelvet	Short reads	De Bruijn graph	http://metavelvet.dna.bio.keio.ac.jp
Omega2	Short reads	Overlap layout consensus	https://github.com/qiumingyao/omega2
metaFlye	Long reads	Overlap layout consensus	https://github.com/fenderglass/Flye
Canu	Long reads	Overlap layout consensus	https://github.com/marbl/canu
NECAT	Long reads (Nanopore)	String graph	https://github.com/xiaochuanle/NECAT
HybridSPADES	Hybrid	De Bruijn graph	https://github.com/ablab/spades
OPERA-MS	Hybrid	De Bruijn graph	https://github.com/CSB5/OPERA-MS
HASLR	Hybrid	De Bruijn graph	https://github.com/vpc-ccg/haslr
Wegan	Hybrid	Synthetic scaffolding graph	https://github.com/adigenova/wengan

**Table 5:** Bioinformatic tools for quality control of metagenome assembly. All websites/GitHub Repository links were accessed on 23 December 2022.

Program	Type of Data	Website/GitHub Repository
MetaQuast	Reference-based	http://bioinf.spbau.ru/metaquast
DeepMAsED	Non-reference	https://github.com/leylabmpi/DeepMAsED
REAPR	Non-reference	https://www.sanger.ac.uk/tool/reapr/
CheckM	Non-reference	https://github.com/Ecogenomics/CheckM
BUSCO	Non-reference	https://busco.ezlab.org
VALET	Non-reference	https://www.cbcb.umd.edu/software/valet

**Table 6:** Bioinformatic taxonomic profilers, including algorithm, use of custom databases, and website or GitHub repository. All websites/GitHub Repository links were accessed on 23 December 2022.

Program	Type of Classifier	Allows Custom Databases	Website/GitHub Repository
Kraken2	k-mers	Yes	https://ccb.jhu.edu/software/kraken2/
Kraken-HLL	k-mers	Yes	https://github.com/Krischan/krakenhll
KrakenUniq	k-mers	Yes	https://github.com/fbreitwieser/krakenuniq
Centrifuge	k-mers	Yes	https://github.com/DaehwanKimLab/centrifuge
Ganon	k-mers	Yes	https://github.com/pirovc/ganon
Bracken	k-mers	Yes	https://github.com/jenniferlu717/Bracken
MetaCache	k-mers	Yes	https://github.com/muellan/metacache
CLARK	k-mers	Yes	http://clark.cs.ucr.edu
VirusTaxo	k-mers	No	https://omics-lab.com/virustaxo
Metavir2	k-mers	No	https://github.com/jhayer/metavir
k-SLAM	k-mers	Yes	https://github.com/aindj/k-SLAM
Taxonomer	k-mers	No	http://taxonomer.com
LMAT	k-mers	No	https://computing.llnl.gov/projects/livermore-metagenomics-analysis-toolkit
Sourmash	k-mers	No	https://sourmash.readthedocs.io/en/latest/
metaOthello	k-mers	Yes	https://github.com/xa6xa6/metaOthello
ProPhyle	k-mers	No	https://prophyle.github.io
TaxMaps	k-mers	Yes	https://github.com/nygenome/taxmaps#sge
Kaiju	Protein-coding	Yes	https://kaiju.binf.ku.dk
DIAMOND	Protein-coding	Yes	https://github.com/bbuchfink/diamond
MMseqs2	Protein-coding	Yes	https://github.com/soedinglab/MMseqs2
IGGsearch	Marker gene	No	https://github.com/snayfach/IGGsearch
MetaPhlAn3	Marker gene	No	https://huttenhower.sph.harvard.edu/metaphlan
GOTTCHA	Marker gene	No	https://lanl-bioinformatics.github.io/GOTTCHA/
DeepVirFinder	CNN	No	https://github.com/jessieren/DeepVirFinder
BLAST	Alignment-based	Yes	https://blast.ncbi.nlm.nih.gov/Blast.cgi
DUDes	DUD	No	https://github.com/pirovc/dudes
MCP	Alignment-based	Yes	https://microba.com/microbiome-research/

**Table 7:** Bioinformatic tools to perform metagenomic binning in short-read and long-read datasets or after assembly. All websites/GitHub Repository links were accessed on 23 December 2022.

Program	Read Length	Website/GitHub Repository
MetaBAT	Short reads	https://bitbucket.org/berkeleylab/metabat/src/master/
GroopM	Short reads	https://github.com/centre-for-microbiome-research/GroopM
MaxBin	Short reads	https://sourceforge.net/projects/maxbin/
CONCOCT	Short reads	https://github.com/BinPro/CONCOCT
MyCC	Short reads	https://sourceforge.net/projects/sb2nhri/files/MyCC/
SolidBin	Short reads	https://github.com/sufforest/SolidBin
BMC3C	Short reads	http://mlda.swu.edu.cn/codes.php?name=BMC3C
COCACOLA	Short reads	https://github.com/younglululu/COCACOLA
GraphBin	Short reads	https://github.com/metagentools/GraphBin
METAMVGL	Short reads	https://github.com/ZhangZhenmiao/METAMVGL
VAMB	Short reads	https://github.com/RasmussenLab/vamb
LRBinner	Long reads	https://github.com/anuradhawick/LRBinner
MEGAN-LR	Long reads	http://software-ab.cs.uni-tuebingen.de/download/megan6/megan-lr/
BusyBee Web	Long reads	https://ccb-microbe.cs.uni-saarland.de/busybee

## Data Availability

Not applicable.
